# Analysis of morphologic and morphometric features of the temporomandibular joint in patients with and without temporomandibular disorders

**DOI:** 10.3389/fdmed.2026.1950507

**Published:** 2026-09-29

**Authors:** Shruthi Hegde, Vidya Ajila, Chethan Hegde, Marakini Subrahmanya Ravi

**Affiliations:** 1 Department of Oral Medicine and Radiology, AB Shetty Memorial Institute of Dental Sciences (ABSMIDS), Nitte (Deemed to be University), Mangalore, India; 2 Department of Prosthodontics, AB Shetty Memorial Institute of Dental Sciences (ABSMIDS), Nitte (Deemed to be University), Mangalore, India; 3 Department of Orthodontics and Dentofacial Orthopaedics, AB Shetty Memorial Institute of Dental Sciences (ABSMIDS), Nitte (Deemed to be University), Mangalore, India

**Keywords:** cone beam computed tomography, mandibular condyle, quality of life, temporomandibular disorders, temporomandibular joint

## Abstract

**Background:**

Temporomandibular disorders (TMD) are common craniofacial conditions characterized by pain and functional limitation. The study was conducted to investigate the association of morphology and morphometric features of the temporomandibular joint (TMJ) with TMD using cone beam computed tomography (CBCT).

**Methods:**

This cross-sectional study evaluated 264 TMJs using CBCT of 132 patients within the age range of 18–60 years. The study comprised 66 patients with TMD in the study group and 66 patients without TMD in control group. Parameters analyzed included shape of condyle, glenoid fossa, bony changes, condylar height and width, joint space dimensions, roof of glenoid fossa(RGF) thickness, width and depth. Statistical analysis was performed using SPSS version 26.0. Holm–Bonferroni correction was applied for multiple comparisons. Statistical significance was defined at an adjusted alpha level of <0.05. Effect sizes were calculated for all comparisons.

**Results:**

Females predominated in both the study (66.7%) and control (69.7%) groups. Among TMD patients, 16.7% presented with muscle disorders, 42.4% with disc displacement, and 40.9% with joint disorders. Angle-shaped condyles were more frequent in TMD patients on the left side (*p* = 0.037) and on the right side (*p* = 0.12); however, after adjustment, *p*-values exceeded 0.05. Glenoid fossa morphology showed no significant bilateral differences. Degenerative changes were significantly associated with TMD (*p* < 0.001). The RGF was significantly thinner in cases on the left (*p* = 0.002) and right (*p* = 0.007) sides. On comparison of the means of right and left morphometric parameters between cases and controls, reduced RGF in cases was statistically significant (*p* = 0.008), and other parameters demonstrated no significant differences (*p* > 0.05). Intra-examiner reliability was excellent (ICC > 0.94).

**Conclusion:**

The present morphometric analysis revealed a significant association between reduced RGF thickness and TMDs, warranting further validation in longitudinal studies. Bilateral variations in TMJ morphology underscore the importance of assessing both sides independently, while degenerative changes detected in over one-third of patients highlight the value of CBCT in comprehensive evaluations. Importantly, the normative dataset generated here provides a regional anatomical reference for TMJ prosthesis design and supports CBCT-based evaluation in diagnosis, monitoring, and treatment planning.

## 1 Introduction

Temporomandibular disorders (TMDs) represent a group of common conditions affecting the craniofacial region, frequently associated with chronic pain, joint noise, and functional limitation (1, 2). The prevalence of TMDs has been reported to range between 5% and 31% in the general population, with recent studies indicating an increasing trend among adults (3–6). TMDs constitute the second most frequent cause of orofacial pain after odontogenic conditions (7). Chronic pain is the primary reason for seeking professional healthcare consultation. This condition is also associated with reduced quality of life and increased healthcare costs (8–11).

Temporomandibular disorders are associated with the temporomandibular joint (TMJ), masticatory muscles, and related musculoskeletal structures (12). The TMJ is a specialized synovial joint formed by the articulation of the mandibular condyle with the glenoid fossa, separated by an articular disc (13). The osseous components of the TMJ are dynamic and undergo continuous remodelling even after the completion of growth, influenced by functional demands, mechanical loading, age, sex, and pathological conditions (14). Mechanical overload is a major factor in TMJ degeneration (15). Joint loading deforms cartilage, with the magnitude of deformation and resulting stress determined by the applied force and tissue stiffness. These biomechanical properties are essential because they regulate stress distribution and load absorption, influence extracellular matrix synthesis and cartilage stiffness over time, and alter stress transfer to the subchondral bone, thereby driving both acute injury and long-term remodelling (16).

The multifactorial etiology and complex presentation of TMDs necessitate a comprehensive diagnostic approach, integrating detailed clinical examination with appropriate radiographic evaluation (17). Imaging of the TMJ allows clinicians to assess the integrity and relationships of osseous and soft tissue components. Magnetic resonance imaging (MRI) is preferred for soft tissue imaging of the TMJ (18). Despite its diagnostic value, the high cost and reliance on tertiary care centres for MRI examinations pose barriers to its routine application in dental practice.

Cone beam computed tomography (CBCT) is preferred by dental specialists for evaluating the osseous components of the TMJ due to the diagnostic information provided, lower radiation dose, cost-effectiveness, and availability (19). The TMJ view software feature in CBCT allows visualization of the TMJ in corrected sagittal and coronal sections, accommodating the variable angulation of each joint (19). Previous studies have revealed that CBCT evaluation led to changes in the primary diagnosis and management of patients with TMDs (20). Condylar morphology has been evaluated in various studies involving healthy individuals and other study populations (14, 21–24). Although numerous recent studies have evaluated TMJ morphology, there remains a paucity of comprehensive investigations that integrate clinical data with detailed radiographic evaluation using CBCT. Specifically, no single study has systematically examined the combined influence of mandibular condyle morphology, glenoid fossa architecture, condylar bony alterations, condylar height and width, joint space dimensions (anterior, posterior, and superior), glenoid fossa roof thickness, and fossa width and depth in patients with and without TMDs. The ability to diagnose and/or predict the severity of TMD through morphometric evaluation of TMJ structures on CBCT images would be immensely beneficial to dental practitioners. The objective of the present study was to evaluate and compare the morphology and morphometric features of the TMJ using CBCT in patients with and without TMDs. The research hypothesis was that there are differences in TMJ morphologic and morphometric features between patients with and without TMD.

## 2 Materials and methods

The study was conducted in a dental hospital and research institute. This study was conducted in compliance with the ethical principles outlined in the Declaration of Helsinki and received approval from the University Central Ethics Committee (Approval No. NU/CEC/2024/742). Observational, cross sectional study adhered to STROBE Statement (25).

A standardized protocol was followed to evaluate all new patients attending the dental hospital. Patients were examined by experienced specialists using an approved TMD examination protocol to ensure consistency in clinical assessment. The study was explained to eligible patients, and written informed consent was obtained from those willing to participate. Participants were recruited using convenience sampling from patients attending the outpatient department during the study period.

Patients aged 18–60 years presenting with pain in the TMJ region or muscles of mastication and diagnosed with TMD according to the Research Diagnostic Criteria for Temporomandibular Disorders (RDC/TMD) Axis I were included in the study group (26).

The control group comprised patients aged 18–60 years without clinical signs or symptoms of TMD who underwent CBCT for diagnostic or treatment-planning purposes unrelated to TMJ symptoms, such as implant planning or evaluation of impacted teeth. This approach avoided additional radiation exposure associated with CBCT acquisition for healthy individuals solely for research purposes. To minimize the inclusion of asymptomatic or subclinical TMJ pathology, all control-group CBCT scans were evaluated for radiographic evidence of degenerative TMJ changes, and patients demonstrating such changes were excluded.

Patients with a history of trauma, TMJ surgery, congenital abnormalities, tumours, growths in the orofacial region, systemic arthritis, pregnancy were excluded from both groups. CBCT scans with inadequate image quality or resolution for reliable assessment were also excluded.

Age- and sex-frequency matching was applied between the study and control groups to minimize potential demographic confounding. Control-group recruitment was monitored throughout the study period to maintain an age range and sex distribution comparable to those of the study group.

Based on the Standard deviation of superior space in the normal group 0.73 mm, and the temporomandibular disorders group 1.25 mm, mean difference 0.5 (27) effect size 0.5050 *α* error 5%, power 80% for two-sided test the required sample per group is 66 for two groups total of 132. This is calculated using nMaster software version 2.

The study comprised of a total of 132 patients and evaluated 264 Joints (132 right TMJ and 132 Left TMJ). The control group includes 66 patients (132 TMJ) without TMD and the study group includes 66 patients (132 TMJ) with TMD. A total of 11(3 morphologic and 8 morphometric) parameters were evaluated in each joint.

CBCT analysis was performed for all patients using a Planmeca ProMax 3D Mid unit (Planmeca, Helsinki, Finland) with Planmeca Romexis Viewer software (version 4.6.2R). A standardized positioning and exposure protocol was followed for all scans. Patients were positioned upright, with the Frankfort horizontal plane parallel to the floor and the midsagittal plane perpendicular to it. Scans were acquired with a field of view of 20 × 17.4 cm, voxel size of 0.4 mm, tube voltage of 90 kVp, tube current of 10 mA, and exposure time of 8–13s.

For morphometric assessment of the TMJ, corrected sagittal and coronal sections were generated for each joint. The axial section demonstrating the widest mediolateral dimension of the condyle was first identified, and a line connecting the most medial and lateral poles of the condyle on this section was used as the reference axis for secondary reconstruction. The corrected coronal plane was oriented parallel to this axis, and the corrected sagittal plane perpendicular to it, with a reformatted slice thickness and interval of 1 mm. The sections providing optimal visualization of the condyle, glenoid fossa, and articular eminence were selected for morphometric measurements. The same anatomical landmarks and orientation criteria were consistently applied to all TMJs to ensure reproducibility.

Morphologic features evaluated in the CBCT scan volumes were mandibular condyle shape, glenoid fossa shape, condylar bony changes. Morphometric parameters such as condylar height, condylar width, anterior joint space, posterior joint space, superior joint space, glenoid fossa roof thickness, glenoid fossa width, glenoid fossa depth were evaluated in the CBCT. The condylar bone shape was assessed in the coronal section as convex, flat, angled and round. Glenoid fossa shapes were assessed in the sagittal plane as either oval, round, trapezoidal and triangular. Condylar bony changes were recorded as normal, flattening, surface erosions, Ely cyst, sclerosis, osteophytes, and loose joint body.

Morphometric evaluation of the TMJ was carried out as described by Al-koshab et al. (24). A sagittal section with a clear condyle and glenoid fossa appearance was selected. Condylar height was determined as the perpendicular distance from the most superior point of the condyle to a reference line drawn between the lowest point of the sigmoid notch and perpendicular to the tangent of the posterior border of the mandibular ramus. In the coronal section, the medial and lateral poles were identified at the regions of greatest convex curvature of the condylar surface. The distance between lateral and medial poles was measured for condylar width.

Linear measurements of the joint spaces between the mandibular condyle and the glenoid fossa were obtained. The superior joint space (SJS) was defined as the distance between the highest point of the condyle and the deepest portion of the glenoid fossa. The anterior joint space (AJS) was measured from the most anterior aspect of the condylar head to the corresponding point on the glenoid fossa, while the posterior joint space (PJS) was assessed between the most posterior aspect of the condylar head and the glenoid fossa.

In the sagittal plane, glenoid fossa thickness was assessed as the distance between its outer and inner cortical outlines. The width of the fossa was defined as the linear distance from the lowest point of the articular eminence to the posterior glenoid margin. Fossa depth was measured as the perpendicular distance from the highest point of the fossa to a baseline drawn through the lowest point of the articular eminence and the posterior glenoid process.

All measurements were performed by a trained maxillofacial radiologist who was blinded to the participants' group allocation (study/control) during image assessment to minimize measurement bias. Measurements were repeated for 20 CBCT scans after a 2-week interval to evaluate intra-observer reliability.

Data were analyzed using SPSS for Windows (version 26.0; IBM Corp., Armonk, NY). Descriptive statistics for qualitative variables were presented as frequencies and percentages, while quantitative variables were expressed as mean and standard deviation. Between-group comparisons for continuous data were performed using the unpaired t-test or the Mann–Whitney U test, as appropriate. Categorical variables were compared using the Chi-square test. To control for Type I error, the Holm–Bonferroni correction was applied for multiple comparisons across both continuous and categorical data. Statistical significance was defined at an adjusted alpha level of <0.05. Effect sizes were calculated for all comparisons: Cramer's V for categorical variables, Cohen's d for continuous variables with parametric distributions, and rank biserial correlation for non-parametric continuous variables. Results were summarized using graphs and tables. Intra-observer reliability was assessed using the intra-class correlation coefficient (ICC), with values >0.70 considered acceptable.

## 3 Results

Patients reporting to the Department of Oral Medicine and Radiology were screened for eligibility according to the predefined inclusion and exclusion criteria. A total of 132 participants were enrolled in the study. Of these,66 patients diagnosed with TMD were assigned to the study group, while 66 patients without TMD were allocated to the control group. The study evaluated 264 Temporomandibular joints (132 right TMJ and 132 Left TMJ) using CBCT.

Study found that 66.7% of study group patients were females and 33.3% were males. Similarly, 69.7% of control patients were females and 30.3% were males. The mean age of study group patients was 31.1 ± 11.5 years and mean age of control group patients was 35.8 ± 11.51 years.

In the study group,16.7% of patients had muscle disorders,42.4% had disc displacements disorders, and 40.9% had joint disorder such as arthralgia, arthritis, arthrosis.

The percentage of type of TMD was comparable between males and females. Distribution of study group patients according to gender (Table 1), was analysed using the Chi-Square test.

**Table 1:** Distribution of cases with TMD according to gender.

	Muscle disorders	Disc displacements	Arthralgia, arthritis, arthrosis	Total	*p* value
**Gender**	***N* (%)**	***N* (%)**	***N* (%)**	***N* (%)**	
Males	4 (18.2)	9 (40.9)	9 (40.9)	22 (100)	*p* = 0.96
Females	7 (15.9)	19 (43.2)	18 (40.9)	44 (100)	NS

N, number; %-percentage; NS, not significant.

The intra-examiner reliability was assessed for a small percentage of the sample. The intra-class correlation coefficient was used to determine the agreement between the same examiner at different time intervals. A value more than 0.70 was considered reliable. The analysis revealed excellent intra-examiner agreement (>0.94).

The study evaluated the shape of the mandibular condyle across all 264 TMJs of the 132 participants. The association between condylar shape among study patients and controls was analyzed (Table 2).

**Table 2:** Association between shape of condyle among study and control patients.

Shape of Condyle	Right					Left				
	**Study group**	**Control group**	**Total**	**Effect size (Cramer’s V)**	***p* value**	**Study group**	**Control group**	**Total**	**Effect size (Cramer’s V)**	***p* value (*p* adjusted)**
	***N* (%)**	**N (%)**	***N* (%)**			***N* (%)**	***N* (%)**	***N* (%)**		
Angled	13 (19.7)	3 (4.5)	16 (12.1)	0.233	*p* = 0.128 (0.128) NS	14 (21.2)	4 (6.1)	18 (13.6)	0.253	*p* = 0.037 (0.074) NS
Convex	30 (45.5)	35 (53)	65 (49.2)			23 (34.8)	34 (51.5)	57 (43.2)		
Flat	14 (21.2)	17 (25.88)	31 (23.5)			21 (31.8)	23 (34.8)	44 (33.3)		
Round	8 (12.1)	10 (15.2)	18 (13.6)			8 (12.1)	5 (7.6)	13 (9.8)		
Others	1 (1.5)	1 (1.5)	2 (1.5)			0	0	0		
Total	66 (100)	66 (100)	132 (100)			66 (100)	66 (100)	132 (100)		

N, number; %-percentage; N, not significant.

On the right side, 19.7% of study patients exhibited angled condyles compared to 4.5% of controls. Convex condyles were observed in 45.5% of study patients and 53% of controls, while flat condyles were present in 21.2% of study patients and 25.9% of controls. The association between angled condyles in the TMD group at the right TMJ was statistically non-significant (adjusted p = 0.128).

On the left side, 21.2% of study patients demonstrated angled condyles compared to 6.1% of controls. Round condyles were observed in 12.1% of study patients and 7.6% of controls, while convex condyles were more frequent in controls (51.5%) than in study patients (31.8%). Although the higher prevalence of angled condyles in the study group reached statistical significance (p = 0.037), the adjusted *p*-value (p = 0.074) indicated a non-significant association.

The study evaluated the shape of the glenoid fossa among 132 participants. Distribution of participants according to shape of glenoid fossa among study group and control group in the right and left TMJ shown in Figure 1. Association between the shape of the glenoid fossa among study and control patients was analysed using the Chi-Square test. There was no statistically significant association between the shape of the glenoid fossa among study and control group in the right side (*p* = 0.09) as well as left side (*p* = 0.97). The study analyzed condylar morphologic changes using CBCT scan volumes (Table 3). Several morphologic alterations of the mandibular condyle were observed within the study group, as depicted in Figures 2–6.

**Figure 1:**
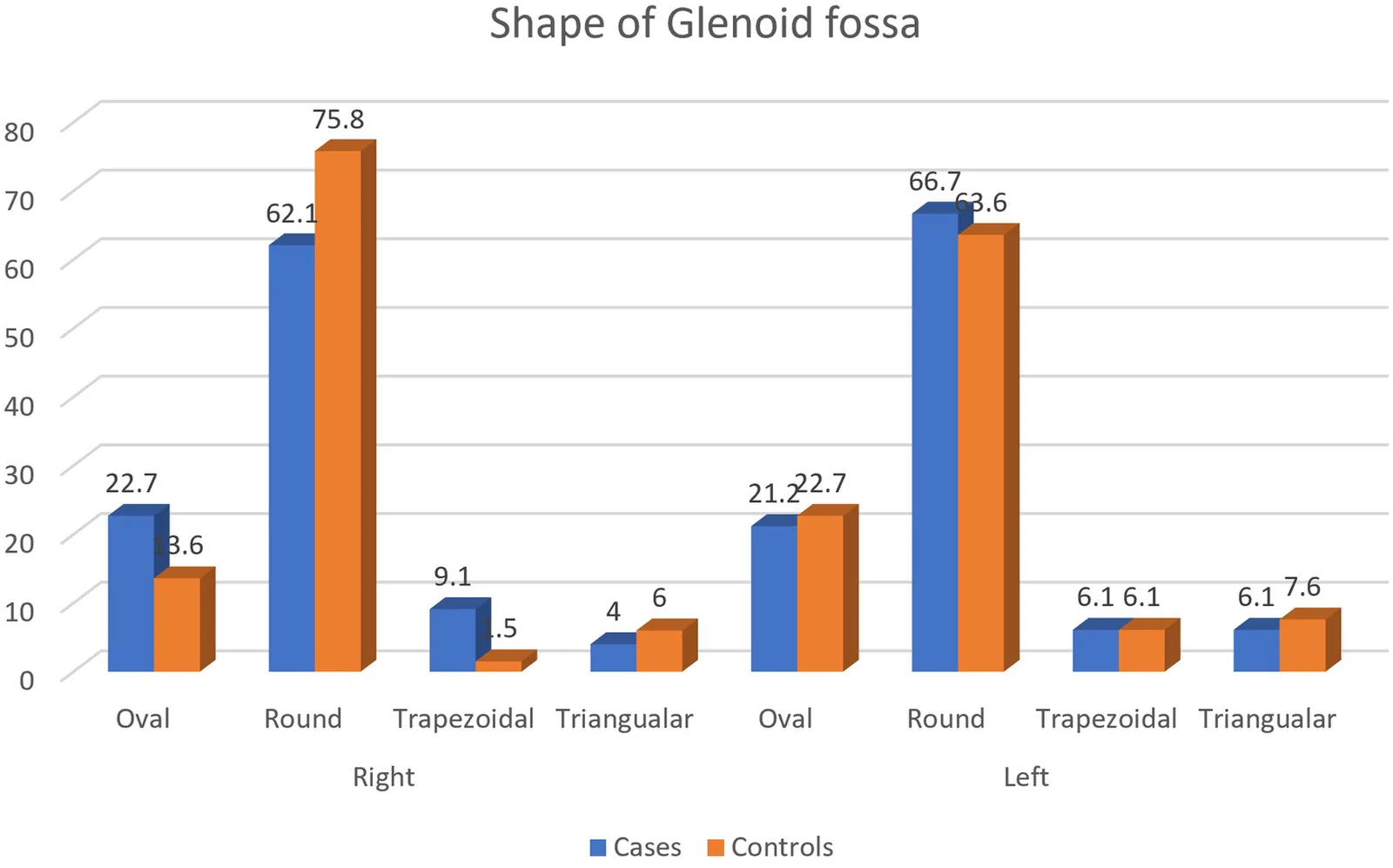
Distribution of participants according to the shape of the glenoid fossa in the study group and control group, assessed separately for the right and left temporomandibular joints.

**Table 3:** Bony changes observed in the mandibular condyle among study and control group.

Parameter	Study group right condyle N (%)	Control group Right condyle *N* (%)	Study group Left condyle *N* (%)	Control group Left condyle *N* (%)
Normal	39 (59.1)	66 (100)	41 (62.1)	66 (100)
Beaking	1 (1.5)	0	2 (3)	0
Loose joint bodies	2 (3)	0	0	0
Erosion	4 (6.1)	0	6 (9.1)	0
Osteophyte	3 (4.5)	0	0	0
Flattening	8 (12.1)	0	6 (9.1)	0
Sclerosis	4 (6.1)	0	3 (4.5)	0
Erosion and flattening	0	0	2 (3)	0
Erosion and osteophyte	2 (3)	0	2 (3)	0
Subchondral cysts	5 (7.5)	0	4 (6)	0
Total	66 (100)	66 (100)	66 (100)	66 (100)

Values are presented as N, number; %, percentage.

**Figure 2:**
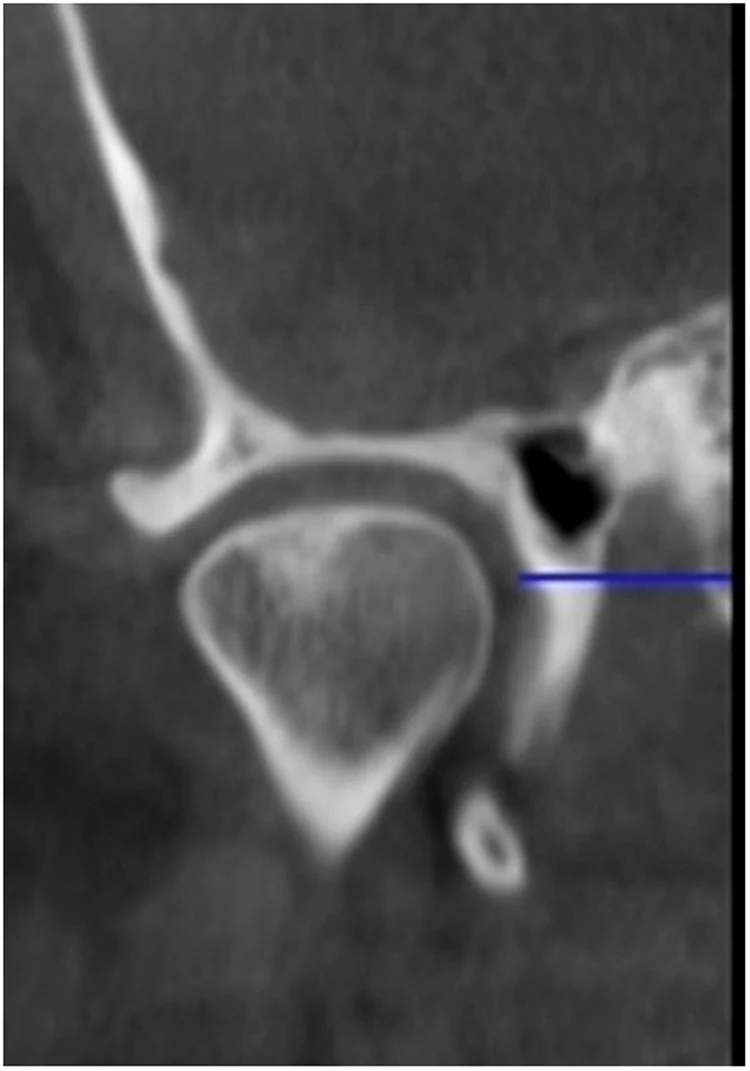
Cone beam computed tomographic (CBCT) image illustrating sclerosis of the mandibular condyle observed in the study group.

**Figure 3:**
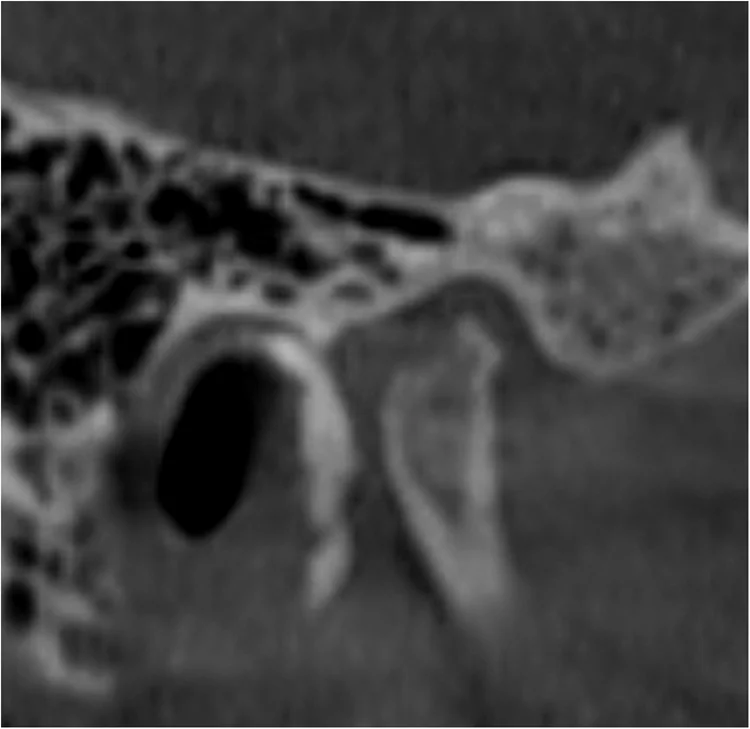
Osteophyte observed in the study group.

**Figure 4:**
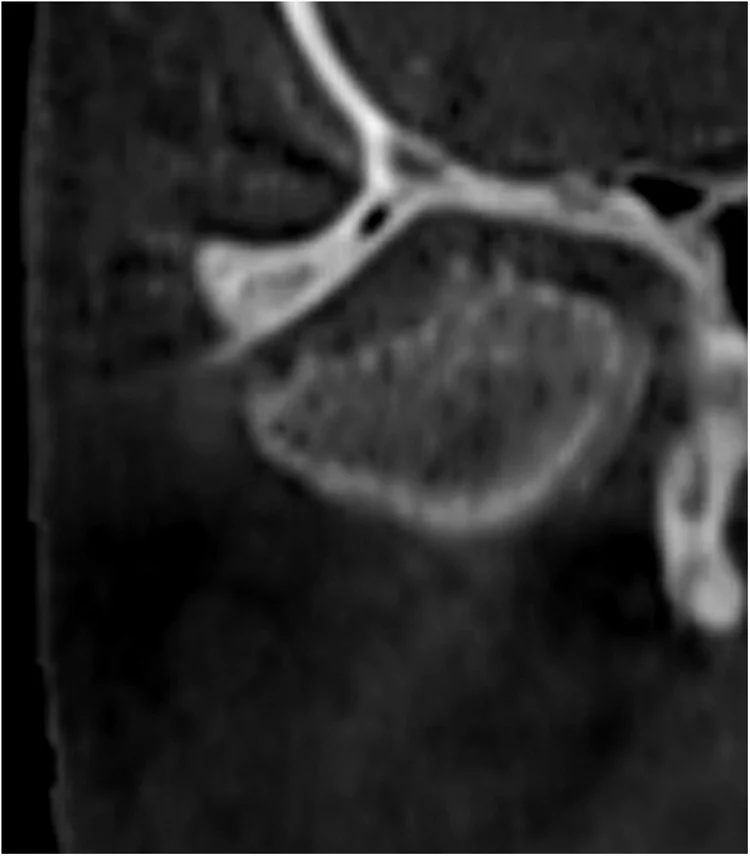
CBCT image showing features of surface erosions.

**Figure 5:**
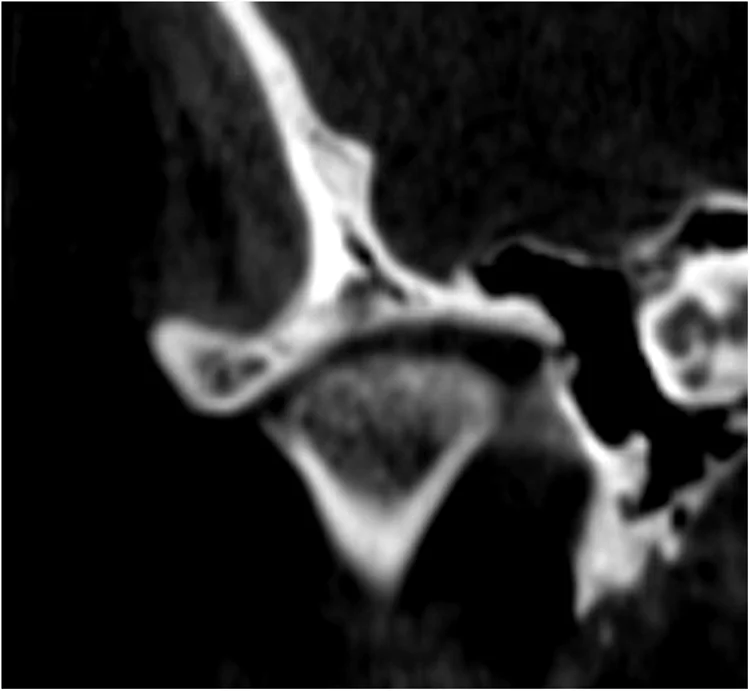
CBCT image demonstrating flattening with erosion.

**Figure 6:**
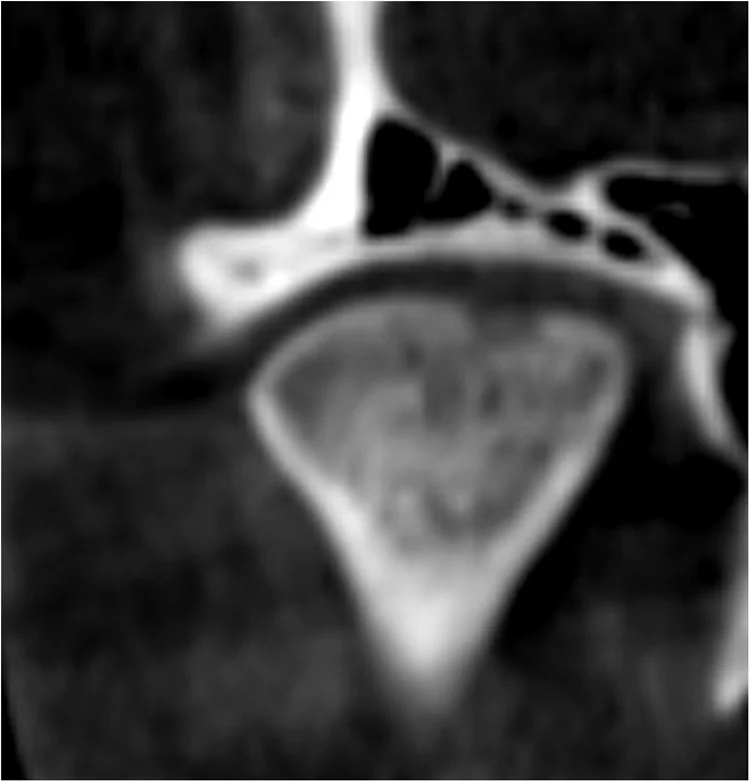
Degenerative features such as subchondral cyst formation shown in the CBCT image.

In the study group, normal condylar morphology was observed in 59.1% of right condyles and 62.1% of left condyles, whereas all condyles in the control group demonstrated normal morphology (100% bilaterally). Pathological variations were noted in the study group, including flattening (12.1% right, 9.1% left), erosion (6.1% right, 9.1% left), sclerosis (6.1% right, 4.5% left), osteophyte formation (4.5% right), and combined lesions such as erosion with flattening or osteophyte changes (up to 3% each). Less frequent findings included beaking, loose joint bodies, and subchondral cysts. Overall, degenerative and morphological changes were confined to the study group, highlighting a statistically significant difference in condylar morphology between cases and controls (p < 0.001).

Morphometric evaluation of the TMJ was conducted, as illustrated in Figures 7–11. Comparison of morphometric values between study patients and controls for both right and left temporomandibular joints was performed (Table 4). Data were expressed as mean ± SD and compared using the unpaired t-test, while median (IQR) values were analyzed with the Mann–Whitney U test. Confidence intervals, effect sizes, and adjusted *p*-values are reported in the table. Analysis of CBCT scan volumes revealed significant morphometric differences between study and control groups with respect to glenoid fossa roof thickness on the left side (p = 0.002) and p adjusted (0.032). In contrast, condylar height, condylar width, anterior joint space, posterior joint space, superior joint space, and the width and depth of the glenoid fossa were comparable between cases and controls bilaterally (p > 0.05).

**Figure 7:**
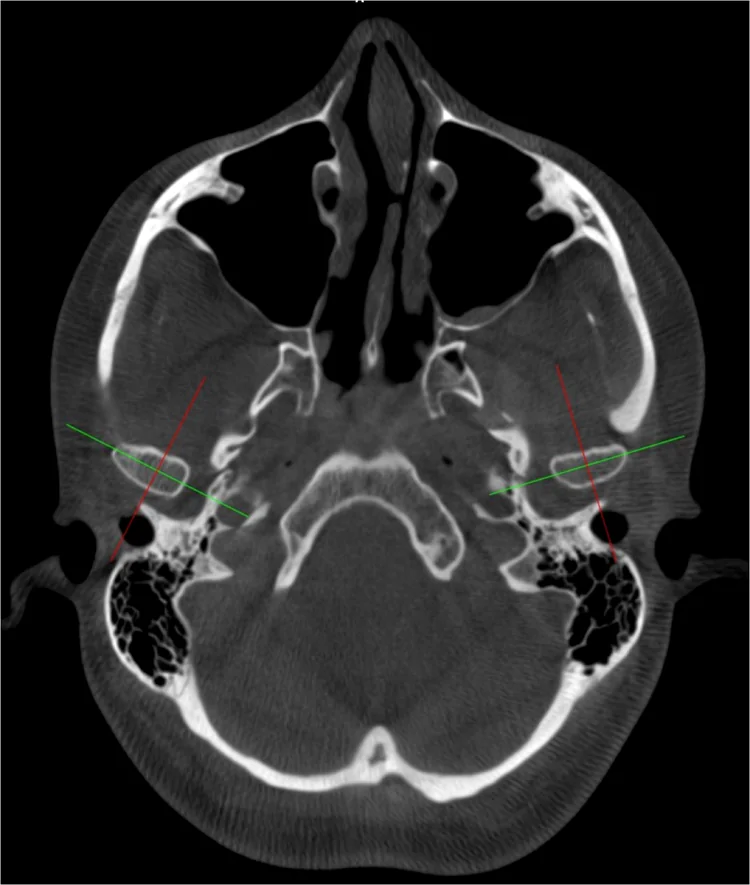
Representative CBCT images showing axial section showing coronal and sagittal reference lines for the TMJ view.

**Figure 8:**
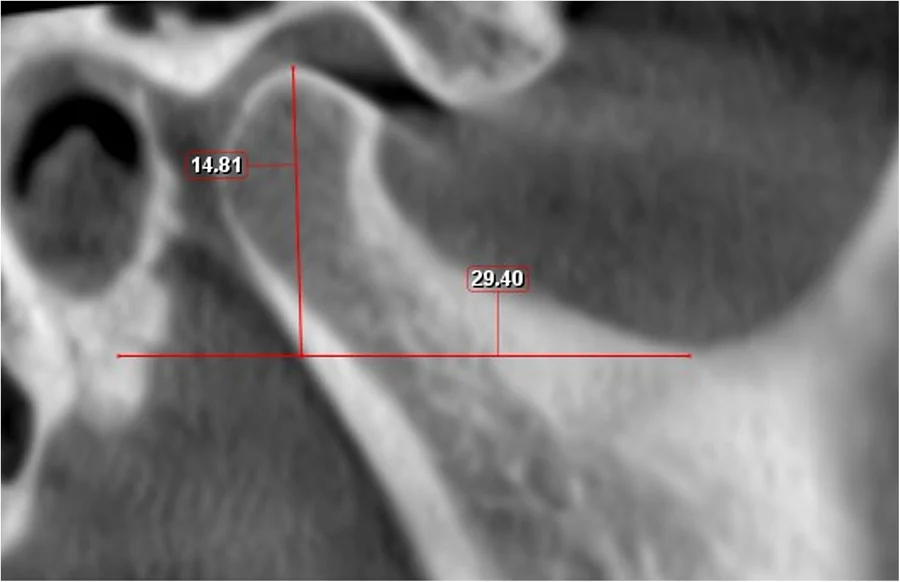
Representative CBCT images showing condylar height measurement.

**Figure 9:**
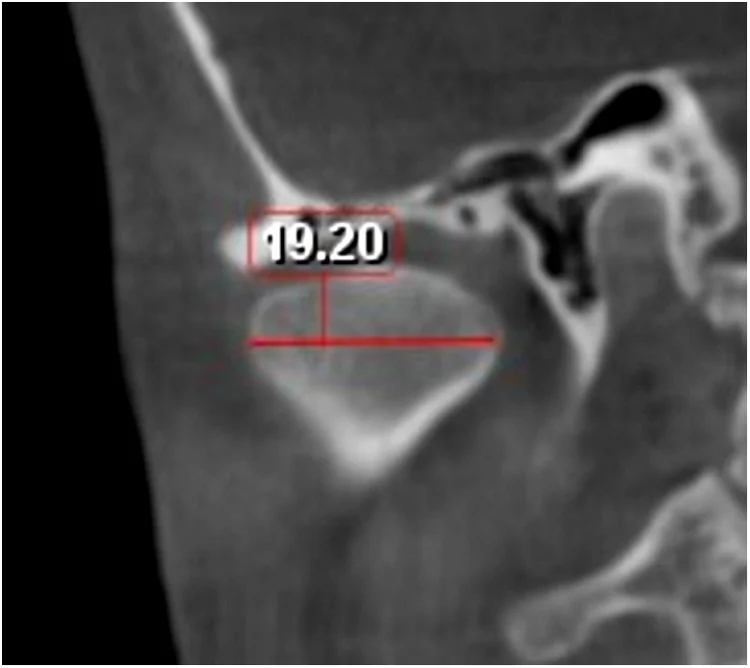
Representative CBCT images showing condylar width assessment.

**Figure 10:**
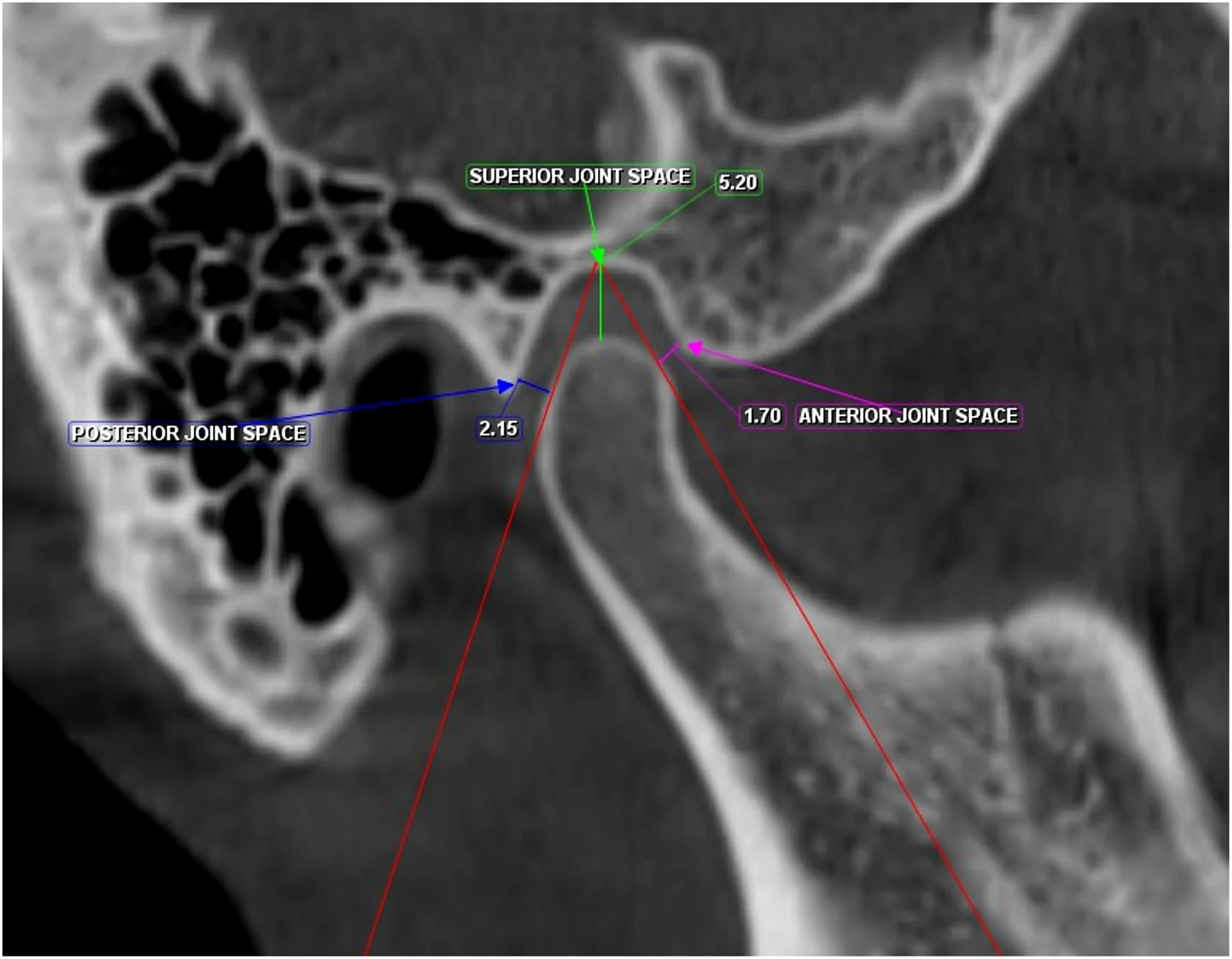
Representative CBCT images showing anterior, posterior, and superior joint space measurements.

**Figure 11:**
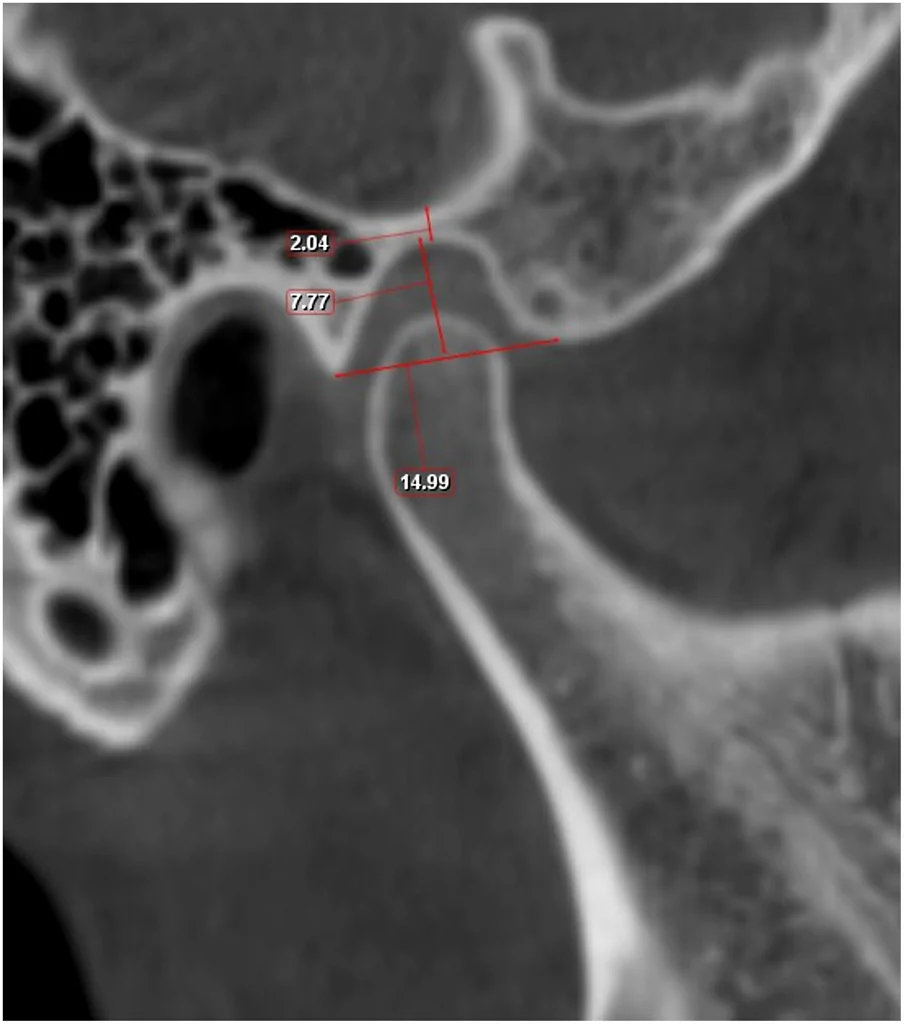
Representative CBCT images showing glenoid fossa roof thickness, glenoid fossa width, and glenoid fossa depth measurement.

**Table 4:** Comparison of morphometric values between study patients and controls – right and left temporomandibular joint.

Parameter	Side	Cases (95% confidence interval)	Controls (95% confidence interval)	Difference (95% confidence interval)	Effect size	*p* value (*p* adjusted)
Condylar height	Right	18.8 ± 3.5 (17.83,19.58)	17.8 ± 3.7 (16.86, 18.70)	1.083 (-.180,2.346)^a^	d = 0.30	*p* = 0.092 (1.000)
	Left	18.5 ± 3.8 (17.49, 19.48)	17.9 ± 3.7 (16.92,18.77)	0.634 (-.689,1.957)^a^	d = 0.17	*p* = 0.345 (1.000)
Condylar width	Right	17.01 ± 2.6 (16.36, 17.68)	16.25 ± 2.3 (15.74, 16.97)	0.756 (-.132 1.644)^a^	d = 0.29	*p* = 0.095 (1.000)
	Left	16.7 ± 2.2 (16.14,17.34)	16.5 ± 2.2) (16.09,17.25)	0.076 (-.741.892)^a^	d = 0.03	*p* = 0.855 (1.000)
Ant. joint space	Right	2.40 (2- 3.3) (2.15, 2.80)	2.35 (2–2.9) (2.15, 2.70)	0.100(-0.150,0.400)^b^	r = 0.09	*p* = 0.363 (1.000)
	Left	2.60 (2.1–3.5) (2.40, 2.80)	2.58 (2–3.4) (2.40, 2.85)	0.000 (-0.280, 0.300)^b^	r = 0.02	*p* = 0.872 (1.000)
Post. joint space	Right	2.43 (2–3.2) (2.30, 2.80)	2.7 (2.3–3.3) (2.50, 2.80)	-0.250 (-0.500,0.000)^b^	r = 0.18	*p* = 0.080 (1.000)
	Left	2.6 (2.1–3.22) (2.50, 2.68)	2.8 (2.1–3.2) (2.70, 2.80)	0.000 (-0.300,0.300)^b^	r = 0.01	*p* = 0.897 (1.000)
Sup. joint space	Right	3.20 (2.4–4) (2.80, 3.40)	3.2 5 (2.4–4.) (2.87, 3.61)	-0.100 (-0.500, 0.300)^b^	r = 0.07	*p* = 0.501 (1.000)
	Left	3.4 (2.6–4) (3.20, 3.60)	3.6 (2.8–4.2) (3.25, 3.60)	-0.100 (-0.500,0.200)^b^	r = 0.07	*p* = 0.459 (1.000)
Glenoid fossa roof thickness	Right	1.80 (1.3–2.4) (1.60, 2.00)	2.15 (1.6–3.2) (2.00, 2.43)	-0.400 (-0.800,-0.500)^b^	r = 0.27	*p* = 0.007 (0.105)
	Left	1.65 (0.8–1.6) (1.60, 2.00)	2.0 (1.6–2.8) (1.85, 2.40)	0.450 (-0.800,-0.100)^b^	r = 0.30	*p* = 0.002 (0.032)*
Glenoid fossa width	Right	13.70 (12.6–14.8) (13.40,14.40)	14.0 (12.8–16.1) (13.65,14.85)	−0.400 (-1.200, 0.400)^b^	r = 0.10	*p* = 0.329 (1.000)
	Left	14.0 (12.2–15.5) (13.75, 14.10)	14.1 (12.5–16.6) (13.55, 14.70)	−0.400 (-1.280. 0.400)^b^	r = 0.10	*p* = 0.338 (1.000)
Glenoid fossa depth	Right	6.0 (5–6.8) (5.36,6.30)	5.3 (4.5–6.6) (5.00, 5.62)	0.400 (-0.100, 0.900)^b^	r = 0.15	*p* = 0.137 (1.000)
	Left	5.60 (4.9–6.6) (5.26,6.00)	5.68 (5–6.7) (5.60, 6.00)	0.000 (-0.460, 0.400)^b^	r = 0.02	*p* = 0.859(1.000)

Values are presented as Mean ± sd compared using an unpaired t-test; Median (IQR) compared using the Mann–Whitney U test. d, Cohen’s d; r, rank biserial correlation.

*Statistically significant using Man-Whitney U test.

a Mean difference.

b Hodges Lehmann median difference.

To facilitate clinical interpretation, the mean morphometric parameters (average of right and left sides) were compared between the study group and controls to obtain a reference value (Table 5). All parameters were comparable between groups except the thickness of the roof of the glenoid fossa (RGF), which was significantly reduced in study patients compared with controls (*p* = 0.001) and p adjusted (0.008).

**Table 5:** Comparison of morphometric parameters (mean of right and left) between cases and controls.

Parameter	Cases Mean ± S.D. (95% Confidence Interval)	Controls Mean ± S.D. (95% Confidence Interval)	Difference (95% Confidence Interval)	Effect size	*p* value (*p* adjusted)
Condylar height Mean ± S.D.	18.605 ± 3.272 (17.76,19.42)	17.813 ± 3.460 (16.96,18.66)	0.79144 (-0.378,1.960)^a^	d = 0.28	*p* = 0.1830 (1.000)
Condylar width; (mean ± sd)	16.926 ± 2.307 (16.30,17.45)	16.510 ± 2.286 (15.94,17.07)	0.41591 (-0.375, 1.207)^a^	d = 0.31	*p* = 0.300 (1.000)
Ant. Joint Space; median (IQR)	2.599 (2.2–3.2) (2.40,2.80)	2.600 (2.1–3.1) (2.40,2.67)	0.090 (-0.150, 0.335)^b^	r = 0.06	*p* = 0.505 (1.000)
Post. Joint Space; median (IQR)	2.649 (2.1–3.3) (2.39, 2.80)	2.600 (2.4–3.2) (2.51,2.84)	-0.100 (-0.350, 0.150)^b^	r = 0.08	*p* = 0.388 (1.000)
Sup. Joint Space; median (IQR)	3.350 (2.68–3.8) (3.20,3.54)	3.400 (2.6–4.08) (3.27,3.45)	−0.150 (-0.490, 0.170)^b^	r = 0.08	*p* = 0.387 (1.000)
Glenoid fossa roof thickness; median (IQR)	1.750 (1.25–2.25) (1.62,1.90)	2.112 (1.6–3.2) (1.88,2.46)	−0.475 (-0.800, -0.200)^b^	r = 0.29	*p* = 0.001 (0.008)*
Glenoid fossa width; median (IQR)	13.650 (12.8–14.8) (13.20, 14.21)	14.300 (12.8–15.9) (13.40,16.52)	−0.375 (-1.095, 0.300)^b^	r = 0.10	*p* = 0.264 (1.000)
Glenoid fossa depth; median (IQR)	5.800 (4.8–6.3) (5.65,5.86)	5.500 (4.5–6.6) (5.27,5.92)	0.150 (-0.255, 0.575)^b^	r = 0.06	*p* = 0.469 (1.000)

SD, standard deviation (unpaired t-test); IQR, interquartile range (Mann–Whitney U test). d, Cohen’s d, r, rank biserial correlation.

*Statistically significant.

a Mean difference.

b Hodges Lehmann Median Difference

## 4 Discussion

The study revealed a female predominance, with 66.7% of patients diagnosed with TMD being women. These findings are consistent with previous reports (28, 29), which attribute this predominance to estrogen-related mechanisms. Clinical evidence indicates that symptoms and signs of TMD tend to increase during pubertal development (30). LeResche et al. reported that TMD pain in women is greatest during periods of lowest estrogen, while rapid fluctuations in estrogen levels may also exacerbate pain (31). Similarly, Berger et al. evaluated the association between estrogen levels and TMD in humans, concluding that estrogen may modulate pain processing in TMD through differential mechanisms (32).

The mean age of patients in the study group was 31.1 ± 11.5 years, indicating that TMD is most commonly observed in adults. The study design included only individuals between 18 and 60 years of age. This age restriction was implemented to minimize confounding factors related to developmental changes during adolescence and degenerative changes in older age groups. Zieliński et al. reported that the reduced variability within the 18–60 year age range may account for the more stable and consistently higher TMD prevalence observed across multiple studies (33). By focusing on this adult age group, the present study ensured that morphometric variations were attributable to TMD pathology rather than age-related remodelling processes. A large retrospective analysis similarly reported a comparable mean age among participants, supporting the observations of the present study (29).

In the present study, the majority of participants were diagnosed with disc displacement disorders (42.4%), followed closely by joint disorders such as arthralgia, arthritis, and arthrosis (40.9%). Muscle-related disorders accounted for only 16.7% of cases. These findings suggest that intra-articular pathologies constitute the predominant etiology of TMD in this cohort, whereas extra-articular conditions such as muscle disorders represent a smaller proportion. This distribution is consistent with previous reports (4).

Mandibular condylar shape distribution was evaluated in 132 TMJs from the study group and 132 TMJs from the control group. Convex morphology was the most frequent shape in the study group, followed by flat, angled, and round forms. The control group also predominantly exhibited convex and flat morphologies; however, a notable difference was that round shape was less common, while angled morphology was the least prevalent. A greater proportion of patients with TMD exhibited angled condylar morphology relative to controls, with a statistically significant difference observed on the left side (p = 0.037) and a non-significant difference on the right side (p = 0.12). However, following adjustment for multiple comparisons using the Holm–Bonferroni method, the observed association between angled condylar morphology and TMD did not remain statistically significant, underscoring the need for cautious interpretation. Angled condylar morphology predominance in TMD patients has been reported in only a few recent studies (34, 35). Çamlıdağ et al. reported that alterations in the shape and size of the mandibular condyle were associated with anterior disc displacement (34). Hu et al. noted that the condyle exhibited a triangular shape in the TMD group and an oval shape in the control group, with statistically significant results (35). Notably, their study included only 20 subjects per group, whereas the present study comprised 66 patients in each group, providing a larger comparative dataset.

Most research has focused on condylar morphology, with limited data available regarding glenoid fossa shape in normal and TMD patients (22). In the present study, assessment of glenoid fossa morphology revealed no significant differences between patients with and without TMD, indicating no association between fossa shape and disease status. The predominant morphology observed in both study and control groups was round, followed by oval, triangular, and trapezoidal. Choudhary et al. evaluated condylar and glenoid fossa shapes along with articular eminence morphology, and similarly reported no significant association between TMJ complex anatomy and TMJ disorders (23). Al-Koshab et al. investigated morphologic variations of the mandibular condyle and glenoid fossa among Malay and Chinese Malaysians in a retrospective study and found no significant differences between the two ethnic groups (24).

This investigation demonstrated that features of degenerative bony changes were limited to study group. Overall, 40.9% of right TMJ and 37.9% of the left TMJ showed degenerative bony changes in CBCT. Commonly occurring bony changes were flattening followed by erosion, sclerosis, osteophyte formation. Loose joint bodies, and subchondral cysts were less common in our cohort. The findings of the present study are in agreement with earlier reports (36, 37). The higher prevalence of flattening and erosion in particular may reflect early degenerative changes, whereas findings such as osteophytes and subchondral cysts indicate more advanced remodelling (36). These results highlight the importance of radiographic evaluation in detecting subtle condylar changes that may not be clinically apparent. Previous studies also suggested that CBCT can be a useful predictor in the assessment of treatment outcomes of disorders affecting the TMJ (38). Mechanical loading of condylar head and glenoid fossa is considered as a key factor for the development of TMJ degenerative changes. It was reported that under normal functional conditions, joint loading supports cartilage maintenance and repair. However, when abnormal forces are applied, the balance can be disrupted, predisposing the joint to degenerative pathology (39).

This study demonstrated morphometric differences between cases and controls. Condylar width was greater in cases on the right side, while the RGF was thinner bilaterally in the study group. The mean thickness of RGF in our study population was 2.1 mm in the control group, whereas Honda et al. reported a mean RGF thickness of 0.8 mm in a Japanese population, and Soydan et al. reported a similar value of 0.84 mm in Turkish patients (40, 41). Adequate thickness of the RGF is important to prevent dislocation of the mandibular condyle into the middle cranial fossa in the event of trauma (41). On comparison of morphometric parameters (mean of right and left) between cases and controls, our study found significantly thinner RGF in cases than in controls. A study by Rosado et al. reported thinner RGF in edentulous patients compared with dentate patients in the presence of flattening and erosion (42). Khojastepour et al. reported that despite increased RGF thickness in most TMD patients, a higher prevalence of RGF discontinuity was detected in TMD patients (43); in our study population, only reduced RGF thickness was noted in TMD patients, without a corresponding increase in discontinuity. Our study used a 0.4 mm voxel size, whereas Khojastepour et al. used a 0.3 mm voxel size, which may partly account for this difference in findings.

Previous studies have suggested that RGF thickness may vary with the stage of osteoarthritis, thickness may be unaffected in early-stage changes such as flattening and erosion, whereas increased thickness is more typical of later-stage changes such as sclerosis, osteophyte formation, and pseudo cyst formation (40–42). In our study population, features of sclerosis and pseudo cyst were seen in only 4.5% and 6% of patients, respectively, while the majority had disc displacement disorders, early-stage osteoarthritic changes, or muscle disorders. These differing results may be attributable to several factors, including variation in study population, disease stage at the time of imaging, dentition status, patient age, differences in voxel size and imaging protocols (40–42). The clinical relevance of markedly thin RGF in TMD cases remains uncertain and warrants further investigation.

Other parameters such as condylar height, joint spaces, and glenoid fossa dimensions were comparable between groups. The wider condylar dimensions observed in cases may be associated with altered loading conditions or pathological stress. As this study is cross-sectional, no causal or adaptive relationship can be established.

The study showed morphologic and morphometric variations in the right and left side of same patient. Asymmetry between right and left TMJ was reported in previous studies (14, 44).

To enhance clinical interpretability, mean morphometric parameters (averaged across right and left sides) were compared between the study and control groups, thereby establishing reference values. These reference value help in patient management and future research.

The strength of the present study lies in its comprehensive evaluation of patients using the well-established RDC/TMD diagnostic protocol in conjunction with CBCT imaging. This integrated approach facilitated a detailed assessment of both morphologic and morphometric parameters of the TMJ. By comparing patients with TMDs to an equal number of controls, the study provides reference values for these parameters.

This study has several limitations. Exact age- and sex-matching between groups could not be achieved, as the control group primarily comprised patients undergoing CBCT for diagnostic or treatment purposes, thereby avoiding intentional radiation exposure. Recruiting healthy volunteers solely for research is ethically unjustifiable; hence, control patients were selected after careful clinical and radiographic evaluation to exclude features of TMD. However, this approach may have introduced selection bias, as the inclusion of clinically asymptomatic, radiographically normal individuals could inflate observed associations. Moreover, the single-centre hospital outpatient population may limit the diversity of participants and reduce the generalizability of findings. The cross-sectional design further restricts causal inference between TMJ morphology and TMDs. In addition, while CBCT provides detailed osseous assessment, it lacks soft tissue visualization, limiting functional correlation. Future studies employing longitudinal designs, rigorously matched control groups, larger sample sizes, and multimodal imaging approaches are warranted to enhance validity and generalizability.

## 5 Conclusion

The present morphometric analysis demonstrated significant associations between reduced glenoid fossa roof thickness with TMDs. Notable morphologic and morphometric variations were also observed between the right and left TMJs within the same patient, underscoring the importance of bilateral assessment. More than one-third of affected individuals exhibited degenerative bony changes detectable on CBCT, highlighting the value of advanced imaging in the comprehensive evaluation of TMDs. RGF thickness should not yet be used as a diagnostic marker until validated longitudinally. Future research incorporating machine learning–based TMJ morphometry may enhance generalizability through standardized assessment across larger populations. This study provides a normative morphometric dataset for the examined population and may serve as a useful anatomical reference for the design of TMJ prostheses suited to patients from this region. Overall, study findings provide reference values for future CBCT-based morphometric assessment of the TMJ, which may support the diagnosis, monitoring, and treatment planning of patients with TMDs.

## Data Availability

The original contributions presented in the study are included in the article/Supplementary Material, further inquiries can be directed to the corresponding author.
